# Risk stratification according to genotype and effect of thromboprophylaxis on obstetric outcomes in women with antithrombin deficiency

**DOI:** 10.1016/j.rpth.2026.106637

**Published:** 2026-05-08

**Authors:** Björn Diemer, Nina Iversen, Lana Othman, Eva-Lotta Hempel, Roza Chaireti, Katarina Bremme

**Affiliations:** 1 Department of Women’s and Children’s Health, Karolinska Institutet, Stockholm, Sweden; 2 Department of Medical Genetics, Oslo University Hospital and University of Oslo, Oslo, Norway; 3 Department of Hematology, Karolinska University Hospital, Stockholm, Sweden; 4 Department of Molecular Medicine and Surgery, Karolinska Institutet, Stockholm, Sweden; 5 Department of Medicine, Solna, Karolinska Institutet, Stockholm, Sweden; 6 Women’s Health, Medical Unit Pregnancy and Delivery, Karolinska University Hospital, Stockholm, Sweden

**Keywords:** hereditary antithrombin deficiency, low-molecular-weight heparin, pregnancy, venous thromboembolism

## Abstract

**Background:**

Hereditary antithrombin (AT) deficiency is a risk factor for venous thromboembolism (VTE) and for obstetric complications and warrants thromboprophylaxis in certain situations. AT deficiency is divided into subtypes with different risk profiles. There is limited knowledge on the specific risks in pregnant women depending on the subtypes.

**Objectives:**

The study aim was to investigate whether genotyping pregnant women with AT deficiency could aid in determining complication risk and subsequently help to individualize antepartum and postpartum treatment.

**Methods:**

This retrospective cohort study included 43 women with AT deficiency with 106 (61 treated, 40 untreated, and 5 unknown) pregnancies between 1990 and 2022 managed at Karolinska University Hospital. Data were gathered on maternal characteristics, heredity and a history of VTE, thrombophilias, treatment during pregnancy, and maternal and neonatal outcome.

**Results:**

Genotyping of AT deficiency was performed, and the women were subsequently divided into low- (*n* = 26) and high-risk (*n* = 17) groups. Types I, IIPE, and IIRS were considered high risk, and type IIHBS low risk. In the low-risk group, 6 untreated women (24%) developed preeclampsia, compared with none among the treated (*P* = .005). Additionally, there were 5 cases (20%) of intrauterine fetal death among the untreated women in the low-risk group, compared with none among the treated women (*P* = .014). We did not find a statistically significant difference in incidence of VTE or other pregnancy complications between the high-risk and low-risk groups.

**Conclusion:**

Our results suggest that untreated women with type IIHBS have a high risk of pregnancy complications, which might warrant thromboprophylaxis regardless of VTE risk.

## Essentials

• Different subtypes of antithrombin deficiency carry different risk profiles for pregnant women.
• We retrospectively investigated complications in genotyped pregnant women.
• Obstetric complications were more common in untreated women with low-risk genotype.
• Thromboprophylaxis might be warranted even in women with low risk of thrombosis.

## 1 Introduction

Hereditary antithrombin (AT) deficiency is an uncommon thrombophilia, with an incidence of less than 0.2% in the general population [1,2]. It increases the annual risk of venous thromboembolism (VTE), ie, pulmonary embolism and deep venous thrombosis, up to 17-fold compared with nondeficient individuals [3]. Pregnancy is associated with an increased thrombotic risk, with healthy women having a 5-fold increased risk of thrombotic events compared with nonpregnant [4]. AT deficiency further increases the risk for thrombosis in pregnant women, and has been reported even to increase the risk for other obstetric complications such as intrauterine fetal death (IUFD) and preeclampsia by observational studies [[5], [6], [7]].

Hereditary AT deficiency is an autosomal dominant trait classified by whether the defect is quantitative (type I) or qualitative (type II) [8]. Type II is further classified into type IIRS, IIPE, and IIHBS, depending on the mutation and which part of the AT protein is affected [9]. Type IIRS affects the reactive site, type IIPE (pleiotropic) affects both the reactive and the heparin-binding site, and type IIHBS only affects the heparin-binding site. Type I accounts for only 12% of patients with AT deficiency in the general population but for 60% of patients with AT deficiency and thrombosis [10]. Type IIHBS carries a much lower risk (hazards ratio, 0.23) of thrombosis compared with type I, type IIPE, and type IIRS [9]. However, it has been associated with a higher number of pregnancy complications, mainly IUFD and recurrent miscarriage, especially in the patients with the homozygous Budapest 3 variant [11]. The main treatment used for pregnant women with AT deficiency is low-molecular-weight heparin (LMWH) as thromboprophylaxis during pregnancy and the postpartum period, as well as substitution with AT concentrate during childbirth or other high-risk procedures where high-dose LMWH cannot be continued [12].

According to a recent study, including part of our cohort [13], the risk for venous thrombosis was associated with the dosage of LMWH used, but nonthrombotic outcomes were not analyzed. Evidence is scant regarding the impact of specific types of AT deficiency on pregnancy outcomes, since current knowledge is mainly based on small studies and case reports [12]. There is therefore not enough data on the differences in obstetric and neonatal outcomes to guide a personalized approach to treatment in pregnant women. This study aims to investigate whether genotyping could aid in determining risk of complications and lead to individualized treatment. Additionally, we study the outcomes in pregnancies both treated and not treated with thromboprophylaxis to identify differences in outcomes, which could help to elucidate the effect of treatment.

## 2 Methods

### 2.1 Study design and patient population

A retrospective observational cohort study was conducted using a registry for pregnant women with AT deficiency who were followed up and delivered at the Department of Women’s Health, Medical Unit Pregnancy and Delivery, Karolinska University Hospital in Stockholm, Sweden. The data gathered in the registry also included neonatal outcomes and data on the newborn children. The registry was started in 2016 and included pregnancies from 1990 onward. After written consent had been obtained, data for this registry were extracted from the patients’ and their children’s digital medical records.

### 2.2 Study cohort

The study cohort included 43 unrelated women and 125 pregnancies. Of these pregnancies 18 were excluded due to legal abortion and 1 due to triplet pregnancy, already considered a high-risk pregnancy. Women and their pregnancies were included regardless of whether they received treatment with AT concentrate and/or anticoagulants during pregnancy and whether they were diagnosed with AT deficiency before or following the pregnancy. The final cohort consisted of 43 women, with a total of 106 pregnancies. Part of this cohort has been included in a multicenter study [13].

### 2.3 Management

Once the diagnosis of AT deficiency had been confirmed, all pregnancies were managed according to local guidelines in collaboration with the Coagulation Unit at Karolinska University Hospital. The diagnosis was based on laboratory analyses of AT according to the local guidelines, and DNA sequencing analysis was conducted later. The same treatment protocol was used for all pregnancies regardless of whether the genotype was known at the time treatment was initiated or not. Treatment with high prophylactic–dose LMWH in a 2-dose fashion was initiated upon confirmation of pregnancy, with LMWH replacing oral anticoagulants if the patient had previously been receiving such treatment. Anti-FXa (activated factor X) was measured 1 week following initiation of treatment, and LMWH dose was adjusted to achieve a trough level of 0.1 to 0.2 kIU/L. Anti-FXa was measured at least every 4 weeks during pregnancy, and LMWH dose was adjusted as needed. During delivery, LMWH was paused for 24 hours, and the patient instead received infusions of AT concentrate to normalize AT activity (1.0 kIU/L; reference interval, 0.8-1.2 kIU/L). After delivery, injections of AT concentrate were continued for 5 days if needed (to maintain an AT activity of at least 0.7 kIU/L), while the LMWH dose was usually lowered according to local guidelines. No patient received AT concentrate during pregnancy, only peripartum. Patients were treated for an additional 12 weeks following delivery. If the patient was treated with warfarin continuously prior to pregnancy, treatment was resumed no earlier than 1 to 2 weeks postpartum. If the patient was treated with direct oral anticoagulants prior to pregnancy, treatment was reinitiated following lactation stop, until which LMWH was used.

### 2.4 Analysis of AT

AT activity was initially analyzed at the Clinical Chemistry Department, Karolinska University Hospital, Stockholm, Sweden, with a method based on FXa inhibition on a Sysmex instrument (Innovance AT; Siemens Healthineers). If the AT level was low or there was a high clinical suspicion of AT deficiency, further analyses were performed. These analyses often included analyzing AT activity with additional methods and performing an AT antigen test. AT antigen was analyzed by BCS XP (Siemens Healthineers).

Following inclusion in the study, allele variant analysis of the *SERPINC1* gene was performed at the Department of Medical Genetics at Oslo University Hospital, Oslo, Norway. This was done by direct Sanger sequencing of all 7 exons and the exon/intron boundaries, the variants were then searched for in the human gene mutation database to verify AT deficiency type I and to distinguish between the subtypes of AT deficiency type II. For analyzing deletions and insertions in samples with no causative mutation found, multiplex ligation dependent probe amplification was performed (MLPA kit P227; MRC). This process has been previously described [13].

### 2.5 Pregnancy characteristics and outcome measures

Risk stratification was based on genotyping results according to the literature, with type I, IIPE, and IIRS being considered as entailing high risk for thrombosis and type IIHBS being considered as having low risk. The study was retrospective, with the genotyping performed after the pregnancy. Thus, treatment given during pregnancy was the same for all patients with known AT deficiency and according to local guidelines and was not decided based on the results of the genotyping.

In addition to baseline maternal characteristics gathered from the visits to the antenatal clinic, further parameters included were treatment during pregnancy, a personal history of VTE, heredity for VTE (defined as VTE among at least 1 first-grade relative, irrespective of age and whether provoked or not), the reason for the initial thrombophilia investigation, miscarriage, and the presence of other thrombophilias. The standard thrombophilia investigation at Karolinska University Hospital includes factor V Leiden (FVL), protein S, protein C, prothrombin gene mutation (PTG2021A), anticardiolipin antibodies, anti–β_2_-glycoprotein I antibodies, and lupus anticoagulant. Maternal outcomes included VTE during pregnancy and the postpartum period, postpartum hemorrhage (PPH), preeclampsia, and mode of delivery. Preeclampsia was defined according to the older definition as blood pressure of ≥140/90 mm Hg and proteinuria after pregnancy week 20, including HELLP (hemolysis, elevated liver enzymes, and low platelets) [14]. PPH was defined as bleeding >500 mL after vaginal birth or >1000 mL after cesarean section [15]. Neonatal outcomes included were gestational age, birth weight, Apgar score after 5 minutes, preterm birth, neonatal VTE, and IUFD. When gestational age was only known to the week, this was defined as week + 0 days.

### 2.6 Statistical analysis

Descriptive statistics were used to present the basic characteristics of the 2 groups. The statistical analyses used to compare the high-risk and the low-risk groups were Mann–Whitney U-test for continuous variables and Fisher exact test for categorical variables. The effects of the difference between groups were assessed with the median difference and its associated CI from the Mann–Whitney U-test, and the odds and its associated confidence interval from the Fisher exact test, for continuous and categorical variables, respectively. A *P* value of <.05 was considered statistically significant. All statistical analyses were performed with R software (version 4.2.2; R Foundation for Statistical Computing).

### 2.7 Ethics

The study was approved by the Regional Ethics Committee (Stockholm) and conducted in accordance with the Declaration of Helsinki. Written informed consent was obtained from all women prior to inclusion.

## 3 Results

### 3.1 Population characteristics

Our study cohort comprised 43 women with pregnancies between 1990 and 2022, who were categorized in either high- or low-risk groups based on genotype in the *SERPINC1* gene and AT antigen level. One woman had type IIPE, 4 had type IIRS, 12 had type I, and 26 had type IIHBS of whom 16 were type IIHBS Basel variant (Supplementary Table). We identified 5 novel variants, 3 missense and 2 small deletions, which all were causing type I AT deficiency. This resulted in 17 women and 44 pregnancies in the high-risk group and 26 women and 62 pregnancies in the low-risk group.

The characteristics of the study population are presented in Table 1. Age and AT activity at diagnosis were significantly different between groups, although the range in both groups was broad. The median age at diagnosis was 9.5 years lower in the high-risk group compared with the low-risk group (*P* = .004), and the AT activity in the high-risk group were significantly lower than those of the low-risk group (*P* = .007). Neither a personal history of VTE at diagnosis nor heredity was significantly higher in the high-risk group. Most women in the high-risk group (94.1%) were investigated either because of heredity or a personal history of VTE, while the reason of investigation was pregnancy complications in 42.3% of the women in the low-risk group.

**Table 1:** Characteristics of pregnant women with AT deficiency.

Characteristic	Low risk	High risk	Total	Effect^a^	95% CI	*P*
Patients (*n*)	26	17	43	—	—	—
Gravidity^b^	2.0 (2.0, 4.0)	3.0 (2.0, 4.0)	3.0 (2.0, 4.0)	0	−1 to 0	.46
Parity^b^	2.0 (2.0, 2.0)	2.0 (2.0, 3.0)	2.0 (2.0, 2.5)	0	−1 to 0	.335
Age at diagnosis (y)	30.5 (28.2, 33.8)	21.0 (17.0, 28.0)	29.0 (21.0, 32.5)	8	2-13	.004
AT activity at diagnosis^c^ (kIU/L)	0.6 (0.6, 0.7)	0.55 (0.5, 0.6)	0.6 (0.5, 0.6)	0.07	0.02-0.12	.007
FVL	3 (11.5)	4 (23.5)	7 (16.3)	2.31	0.334-18.303	.407
History of VTE at diagnosis	4 (15.4)	7 (41.2)	11 (25.6)	3.72	0.745-21.654	.08
Heredity of VTE	14 (53.8)	11 (64.7)	25 (58.1)	1.555	0.381-6.806	.541
Reason for investigation				0.09	0.002-0.753	.014
History or heredity of VTE	15 (57.7)	16 (94.1)	31 (72.1)			
Pregnancy complication	11 (42.3)	1 (5.9)	12 (27.9)			

Values are *n* (%) or median (Q1, Q3) unless stated.

AT, antithrombin; FVL, factor V Leiden; VTE, venous thromboembolism.

a Odds ratio for categorical values and median difference for numerical values.

b At the end of the latest pregnancy.

c Measured when the patient was not pregnant using a FXa inhibition-based method.

All women were screened for other thrombophilias, and FVL in heterozygous form was by far the most common, accounting for 70% of diagnosed thrombophilias other than AT deficiency. In the high-risk group, there were 5 women with a thrombophilia trait other than AT deficiency, 4 with heterozygous FVL and 1 with heterozygous prothrombin gene mutation, all with a history of VTE at diagnosis. In the low-risk group, there were 4 women with other thrombophilias: 2 with heterozygous FVL, 1 with homozygous FVL, and 1 with anticardiolipin antibodies. None of them had a history of VTE. However, 3 of 4 women with VTE in the low-risk group had VTE in conjunction with combined hormonal contraceptives or pregnancy. Altogether, combined hormonal contraceptives or pregnancy was a risk factor for 9 of 11 VTE prior to diagnosis of AT deficiency. Part of those results were included in the multicenter cohort described in the study by Dybedal et al [13].

### 3.2 Pregnancy characteristics

In Table 2, the baseline values at the beginning of pregnancy are presented. The median age at conception was 2.5 years higher in the high-risk group than in the low-risk group (*P* = .021). A higher percentage of women in the high-risk group received treatment with LMWH than in the low-risk group (*P* = .036). No statistically significant difference in body mass index, blood pressure, or frequency of miscarriage was seen.

**Table 2:** Pregnancy characteristics of women with AT deficiency.

Characteristic	Low risk	High risk	Total	Effect^a^	95% CI	*P*
Pregnancies (*n*)	62	44	106			
Age at conception (y)	29.5 (25.0, 33.0)	32.0 (29.0, 34.8)	30.0 (26.0, 33.2)	−3	−6 to 0	.021
BMI (kg/m^2^)	24.6 (21.6, 28.7)	23.0 (21.9, 24.3)	23.8 (21.6, 27.9)	1.95	−0.43 to 4.32	.109
SBP (mm Hg)	110.0 (104.0, 120.0)	110.0 (100.0, 110.0)	110.0 (100.8, 115.0)	3	0-10	.202
DBP (mm Hg)	65.0 (60.0, 70.0)	65.0 (60.0, 70.0)	65.0 (60.0, 70.0)	0	−2 to 5	.443
Treated for AT deficiency^b^	32 (51.6)	29 (65.9)	61 (57.5)	2.692	1.054-7.307	.036
Miscarriage	6 (9.7)	7 (15.9)	13 (12.3)	1.756	0.464-6.874	.378

Values are *n* (%) or median (Q1, Q3) unless stated.

BMI, body mass index; DBP, diastolic blood pressure; SBP, systolic blood pressure.

a Odds ratio for categorical values and median difference for numerical values.

b Women receiving full thromboprophylaxis according to local guidelines during pregnancy, not including those receiving treatment only after an adverse event.

### 3.3 Maternal outcomes

In Table 3, data on maternal outcomes are presented. Four women (9.1%) in the high-risk group had a VTE during pregnancy or the postpartum period. Two of those women were not receiving treatment at the time of the thrombosis, since they had not yet been diagnosed with AT deficiency. One of them experienced a thrombosis in the first trimester and the other 1 week after a cesarean section. The third woman experienced a thrombosis during pregnancy week 6, after having switched treatment from warfarin to high-dose LMWH prophylaxis. She was treated with warfarin due to multiple previous thromboses and AT deficiency. The fourth woman had discontinued her postpartum thromboprophylaxis 3 weeks prior to VTE diagnosis. In the low-risk group, there were 2 (3.2%) cases of VTE during pregnancy. Both were diagnosed with AT deficiency after these thrombotic events and were therefore not receiving treatment when the VTE occurred. One had a thrombosis in the first trimester, and the other had a thrombosis 4 days after a manual placental extraction.

**Table 3:** Maternal outcomes of women with AT deficiency.

Outcomes	Low risk	High risk	Total	OR	95% CI	*P*
VTE	2 (3.2)	4 (9.1)	6 (5.7)	2.969	0.404-34.276	.23
Preeclampsia including HELLP^a^	6 (10.7)	0 (0)	6 (6.5)	0	0-1.234	.078
PPH^a^	10 (17.9)	8 (21.6)	18 (19.4)	1.59	0.467-5.303	.413
Mode of delivery^b^						.465
Vaginal induced	18 (35.3)	10 (27.8)	28 (32.2)			
Vaginal spontaneous	16 (31.4)	13 (36.1)	29 (33.3)			
Assisted vaginal^c^	1 (2.0)	4 (11.1)	5 (5.75)			
Planned section	7 (13.7)	4 (11.1)	11 (12.6)			
Emergency section^c^	9 (17.6)	5 (13.9)	14 (16.1)			

Values are *n* (%) unless stated.

PPH, postpartum hemorrhage; VTE, venous thromboembolism.

a Percentage of all pregnancies excluding miscarriages.

b Percentage of all live births.

c Including cases that were induced prior to instrumental delivery or section.

No women in the high-risk group were diagnosed with preeclampsia, whereas in the low-risk group, 6 cases (10.7%) were diagnosed (*P* = .078). Of these, 3 (50%) fulfilled the criteria for HELLP syndrome. One of the women with HELLP syndrome delivered in week 32 with emergency section due to aggravation of symptoms. The other 5 delivered vaginally at term, and 1 of them developed eclampsia.

There was no significant difference in occurrence of PPH between the high-risk and low-risk groups (21.6% and 17.9%, respectively; *P* = .413). Three patients bled >2000 mL. In 1 case, this was caused by a uterine rupture during an induced vaginal birth, and in one case, it was due to an adherent placenta during a spontaneous vaginal birth. The cause of the third abnormal bleeding was not determined. The patient with uterine rupture was treated with LMWH, while the 2 others were not.

No significant differences in mode of delivery were seen between the high-risk and the low-risk groups. Part of those results were included in the multicenter cohort described previously [13].

### 3.4 Neonatal outcomes

Of 87 live births, 2 newborns presented with cerebral thrombosis after birth, both born to mothers with type I deficiency and found to have AT deficiency themselves after the age of 6 months. The newborns had an Apgar score of 9 and 10 after 5 minutes, respectively. Both had been delivered using vacuum extraction, 1 at 36 weeks and 1 at 39 weeks.

One newborn in the low-risk group presented with seizures and right-sided thrombotic stroke shortly after vaginal birth, with an Apgar score of 4 after 5 minutes. The newborn was later diagnosed with type II AT deficiency, FVL, and heterozygous prothrombin gene mutation. In Table 4, further data on neonatal outcomes are presented. Only 1 neonate was extremely preterm, born in gestational week 27, without any other known pregnancy complication. Only 1 of the preterm neonates was born to a mother with preeclampsia.

**Table 4:** Neonatal outcomes for children born to women with AT deficiency.

Outcomes	Low risk	High risk	Total	Effect^a^	95% CI	*P*
Gestational age (weeks + days)	38 + 6 (37 + 4, 40 + 0)	38 + 5 (37 + 4, 39 + 5)	38 + 6 (37 + 0, 40 + 0)	1	−6 to 6	.809
Birth weight (g)	3300.0 (2951.0, 3650.0)	3240.0 (2820.0, 3617.5)	3272.5 (2905.0, 3641.2)	75	−150 to 295	.507
Apgar 5	10.0 (9.5, 10.0)	10.0 (10.0, 10.0)	10.0 (9.8, 10.0)	0	0-0	.933
Preterm^b^	9 (17.6)	7 (19.4)	16 (18.4)	1.165	0.327-3.991	.785
IUFD^c^	5 (8.9)	1 (2.7)	6 (6.5)	0.287	0.006-2.718	.397

Values are *n* (%) or median (Q1, Q3) unless stated.

Apgar 5, Apgar score after 5 minutes; IUFD, intrauterine fetal death.

a Odds ratio for categorical values and median difference for numerical values.

b Delivered before week 37 + 0.

c Percentage of all pregnancies excluding miscarriages.

Two of the 6 stillbirths occurred to women in the low-risk group in the second trimester, and both fetuses had congenital malformations, 1 with gastroschisis and 1 with omphalocele. Four stillbirths occurred during the late half of the third trimester, 3 to women in the low-risk group and 1 to a woman in the high-risk group with type IIRS. The stillbirths in the late half of the third trimester were not associated with any known malformations or pregnancy complications.

### 3.5 Outcomes in treated vs untreated pregnancies

In 61 pregnancies, patients received thromboprophylaxis, and in 40 pregnancies, patients were untreated. In 5 pregnancies, the treatment status is unknown. VTE was more common in untreated (10.0%) than in treated women (3.3%), although this difference was not statistically significant (*P* = .21). None of the treated women in the low-risk group developed VTE. Miscarriage was also more common in untreated women, although not statistically significant (*P* = .107). Seven untreated (17.5%) and 4 treated women (6.6%) miscarried. As seen in Table 5, there was a statistically significant difference in preeclampsia between treated and untreated women, with all 6 cases occurring in untreated women (*P* = .002). This difference remained even when taking only the low-risk group into account (*P* = .005). There was no difference in PPH when comparing treated with untreated women (*P* = 1).

**Table 5:** Comparison between treated and untreated pregnancies in women with AT deficiency.

Outcomes	Low risk					High risk					Total				
	Untreated	Treated	OR	95% CI	*P*	Untreated	Treated	OR	95% CI	*P*	Untreated	Treated	OR	95% CI	*P*
Pregnancies (*n*)	25	31				8	26				33	57			
Pre-eclampsia	6 (24.0)	0 (0)	0	0-0.599	.005	0 (0)	0 (0)	0	0 to infinity	1	6 (18.2)	0 (0)	0	0-0.442	.002
PPH	4 (16.0)	6 (19.4)	1	0.199-5.62	1	2 (25.0)	6 (23.1)	0.369	0.022-6.137	.558	6 (18.2)	12 (21.1)	0.88	0.254-3.324	1
IUFD	5 (20.0)	0 (0)	0	0-0.801	.014	1 (12.5)	0 (0)	0	0-12	.235	6 (18.2)	0 (0)	0	0-0.442	.002

Values are *n* (%) unless stated. Pregnancies ending in miscarriage and pregnancies with unknown treatment status are not included.

IUFD, intrauterine fetal death; PPH, postpartum hemorrhage.

All 6 cases of IUFD occurred in women who did not receive treatment. There was a statistically significant difference (*P* = .002), which remained when including only the low-risk group (*P* = .014), in which all except 1 case occurred. In the high-risk group, treatment did not correlate with IUFD (*P* = .235). Of the women with IUFD, all of which had type II AT deficiency, 4 had AT Basel, 1 AT Budapest, and 1 type II RS (high risk).

## 4 Discussion

In this retrospective cohort study, which is one of the largest studies on pregnant women with AT deficiency, we observed a high incidence of preeclampsia and IUFD in pregnant women who did not receive thromboprophylaxis. In treated women, on the contrary, pregnancy complications were uncommon.

Most pregnancies ended in live births, and the miscarriage rate of 12.3% did not exceed that of the general population [16]. However, the rate of IUFD was 6%, which is >15 times that of the Swedish national rate [17]. Interestingly, all cases of IUFD occurred in women not receiving treatment. A previous study by Kovac et al. [11] found IIHBS in homozygous form to be associated with IUFD, leading to 8% birth rate in untreated patients, but did not observe this for the heterozygous type IIHBS, where the birth rate was 94%. The question of whether this finding was affected by treatment was not answered in their study [11]. In the cases of late stillbirths, no potentially associated malformations could be found. Regardless, such associations cannot be ruled out since there have been reports of fetal malformations, for example, vena cava inferior atresia, caused by homozygous AT Budapest 3 variant [18]. In a later report from Bravo-Pérez et al. [19] obstetric complications remain very high despite anticoagulation treatment in homozygote type II HBS. Since our study found a high frequency of IUFD in the more common heterozygous IIHBS, IUFD in AT-deficient women might be more common and, therefore, more clinically relevant than previously thought. Additionally, AT Basel, which was highly prevalent in our cohort and was present in 4 of 6 cases of IUFD, is also highly prevalent in both Denmark [20] and Finland [21], whereas other mutations such as AT Budapest 3 [22] and AT Toyama [23], were found in only 2 of the patients in our cohort [13]. Subsequently, the effect of specific mutations cannot be evaluated.

The incidence of preeclampsia in untreated women was 18.2%, which is clearly higher than the incidence reported in Sweden [24] and globally [25]. All 6 women who suffered from preeclampsia were part of the low-risk group and had not received thromboprophylaxis. Interestingly, beneficial effects of LMWH on the risk for preeclampsia have been shown in both women with and without thrombophilia [26], although other studies have questioned these conclusions [27]. The incidence of premature birth was not significantly different between groups, but still 3 to 4 times greater than in the general population [17]. No known pregnancy complication could account for this high incidence.

Since preeclampsia and IUFD were lower in the group of women who were treated with thromboprophylaxis, the question on the possible protective effect of LMWH is raised. There are studies and reviews which show that LMWH appears to decrease the risk for complications such as preterm birth, low weight at birth, and fetal loss [11,[28], [29], [30], [31]], whereas other studies and meta-analyses show contradicting results, at least partly [32,33]. The results from our study appear to support the protective or positive effect of LMWH; however, no comparative analyses were performed. To our knowledge, there are limited data on the effects of thrombophilia on the placenta, but poor placental circulation secondary to placental thrombosis has been suggested as a cause of pregnancy complications in patients with thrombophilia [34]. More trials, optimally including histological examinations of the placenta, could shed light on this hypothesis.

VTE was uncommon in both groups, with no statistically significant difference. This could be attributed to the fact that almost three-fourths of the women in the high-risk group received treatment with LMWH, and that, even in the predisposed AT-deficient population, VTE is a rare occurrence. Despite that, the total incidence of 5.7% is much higher than that in the general pregnant population, where it is ∼0.05% to 0.2% [35]. When comparing untreated with treated women, the difference in the incidence of VTE was not statistically significant, similarly to other studies. One systematic review on women with AT deficiency not receiving thromboprophylaxis found an absolute risk of VTE of 16.6% (95% CI, 0.0%-45.1%) during pregnancy and the postpartum period, not distinguishing between types I and II [36]. Another study looking only at type I deficiency found an incidence of VTE of 11.6% in those not receiving treatment, and no statistically significant difference when treatment was administered [6]. Regardless of the actual incidence, treatment appears to be crucial for these women, especially in women with high-risk AT deficiency [13].

No significant difference in the incidence of FVL mutation between the 2 groups was observed, but the incidence was higher than the 10% incidence of the general Swedish population for both groups [37], which could be secondary to selection bias. Although a low-risk mutation compared with AT deficiency, heterozygous FVL has been shown to significantly increase the risk of VTE in pregnant women (odds ratio, 6.4; 95% CI, 4.0-9.7) [36]. It has also been implicated in adding to the risk already mediated by AT deficiency [38]. All patients with FVL and a high-risk AT deficiency genotype had been investigated for AT deficiency because of a personal history of VTE, which could support this notion.

The main strength of our study is its large cohort. We have included 106 pregnancies, making this the largest study on pregnant women with genotyped AT deficiency, to our knowledge. Additionally, data from the genetic analyses were available for all women, allowing for a reliable risk stratification. Furthermore, all patients were treated at the same hospital and with the same protocol, ensuring that the results are comparable.

There are some limitations to our study. The study included both untreated and treated women (pregnancies), which has likely led to a lower number of adverse outcomes. Therefore, the power of our study might be too low to find significant differences in risk of VTE and adverse pregnancy outcomes between different types of AT deficiency. Data might also be skewed because of how the patients were diagnosed. The investigation was often initiated following an unfavorable outcome, and women without complications might never be diagnosed. This could lead to an overstatement of the incidence of these complications in the AT-deficient population. Some mutations, such as AT Basel, have an increased prevalence among Scandinavian populations, which can overestimate the effect of a specific mutation, whereas simultaneously exaggerating the effect of the most frequent ones. This can limit the potential for generalization of the results.

Our results suggest that treatment with LMWH and, when necessary, AT concentrate is important for pregnant women with AT deficiency, especially for those with type I, type IIRS, and type IIPE genotypes. Even if the risk for VTE appears lower and might not necessarily advocate treatment in women with type IIHBS without other risk factors, the increased risk for preeclampsia and IUFD as manifesting in the untreated pregnancies might warrant treatment. Although this could theoretically call the necessity of genotyping pregnant women into question, the heterogeneity of the complication patterns and difficulties in predicting outcomes require an investigation as complete as possible, including for genetic factors. In addition, the question of treatment intensity is important. Whether high-dose prophylaxis is required or whether normal dose prophylaxis is enough to decrease the risk of preeclampsia and IUFD in the low-risk group is unknown. With further research into this area, genotyping might become an important tool to aid in determining the risk for every specific patient, and subsequently, personalizing the treatment. Considering the potential risks stemming from inadequate treatment, both to the pregnant woman and the fetus, multicenter, larger studies that could confirm and strengthen the results are imperative.
