# Supplementary material for: Risk stratification according to genotype and effect of thromboprophylaxis on obstetric outcomes in women with antithrombin deficiency
Source: Res Pract Thromb Haemost. 2026 May 8;10(4):106637. doi: 10.1016/j.rpth.2026.106637 (PMC13240814; doi:10.1016/j.rpth.2026.106637)
Supplement: Supplementary Material [file mmc1.docx]

Table S1.

Hereditary antithrombin deficiency confirmed by genotyping of the *SERPINC1* gene at the Department of Medical Genetics at Oslo University Hospital, Oslo, Norway.

| **Women** | **Nucleotide exchange** | | **Exon** | **Amino acid exchange** | **AT antigen level at inclusion (kIU/L)** | | **AT activity level at inclusion (kIU/L)** | | **ATD subtype** |  |
| --- | --- | --- | --- | --- | --- | --- | --- | --- | --- | --- |
|  |  | |  |  |  | |  | |  |  |
| 1 | c.749C>T | | 4 | p.Thr25Olle | 1.10 | | ***0.46*** | | Type II PE |  |
| 2,4,5,8,10,17,18,19,21 | c.218C>T | | 2 | p.Pro73Leu | 1.09 (0.82-1.29)† | | 0.58 (0.48-0.71)† | | Type II HBS, Basel |  |
| 22,28,29,30,39,40,48 |  | |  |  |  | |  | |  |  |
| ***3*** | ***c.726_729del*** | | ***4*** | ***P.Thr243Phefs*40*** | ***0.60*** | | ***0.45*** | | ***Type I*** |  |
| 6 | c.2T>G | | 1 | p.M1? | 0.49 | | 0.59 | | Type I |  |
| 7 | Deletion of exon 6 | | 6 |  | 0.57 | | 0.50 | | Type I |  |
| 9 | No mutation found# | | | | 0.59 | | 0.57 | | Type I |  |
| ***11*** | ***c.510_513del*** | | ***3*** | ***p.Lys171**** | ***0.57*** | | 0.50 | | ***Type I*** |  |
| 12,15,32 | c.1271G>A | | 7 | p.Gly424Asp | 0.99 (0.80-1.15)† | | 0.60 (0.55-0.64)† | | Type II RS, Stockholm |  |
| ***13*** | ***c.653T>A*** | | ***4*** | ***p.IIe218Asn*** | ***0.52*** | | ***0.40*** | | ***Type I*** |  |
| ***14*** | ***c.367A>T*** | | ***2*** | ***p.Lys123**** | ***0.57*** | | 0.59 | | ***Type I*** |  |
| 16,25,38 | c.159C>A | | 2 | p.Cys53* | 0.49 (0.46-0.51)† | | 0.53 (0.42-0.59)† | | Type I |  |
| 20 | c.1332_1333del | | 7 |  | 0.5 | | 0.57 | | Type I |  |
| 26 | c.166C>T | | 2 | p.Arg56Cys | 0.94 | | 0.73 | | Type II HBS |  |
| 27,46 | c.236G>A | | 2 | p.Arg79His | 1.15 (1.13-1.17)† | | 0.58 (0.55-0.60)† | | Type II HBS, Padua |  |
| 31 | c.439A>G | | 3 | p.Thr147Ala | 0.78 | | 0.71 | | Type II HBS |  |
| 33,41 | c.805G>A | | 5 | p.Glu269Lys | 0.73 (0.71-0.75)† | | 0.69 (0.66-0.71)† | | Type II HBS, Truro |  |
| ***37*** | ***c.1386T>A*** | | ***7*** | ***p.Cys462**** | ***0.65*** | | ***0.54*** | | ***Type I?*** |  |
| 42 | c.1246G>C | | 7 | p.Ala416Pro | 1.07 | | ***0.48*** | | Type II PE |  |
| 49,51 | c.391C>T | | 2 | p.Leu131Phe | 1.02 (0.85-1.18)† | | 0.52 (0.50-0.53)† | | Type II HBS, Budapest 3 |  |
| 52 | c.233G>A | | 2 | p.Arg78Gln | 1.18 | | 0.65 | | Type II HBS |  |
| 53 | c.235C>T | | 2 | p.Arg79Cys | 1.29 | | 0.60 | | Type II HBS, Toyama |  |
|  |  | |  |  |  | |  | |  |  |
|  | |  |  | | |  | |  |  |  |
|  | |  |  | | |  | |  |  |  |

Novel mutations identified are listed in bold and cursive; the genotyping has previously been published in Dybedal et al. (1).

# Patient 9 was diagnosed before sequencing after VTE with FV Leiden mutation and AT deficiency (AT enz <0.60 and AT imm <0.60).

† Mean value and ranges.

References

1. Dybedal I, Iversen N, Jacobsen AF, Bjørge L, Chaireti R, Henriksson CE, et al. Hereditary antithrombin deficiency and venous thrombosis in pregnancy-results of a retrospective multicenter study. J Thromb Haemost. 2025; 23:2807-2818
